# Preparation of azachalcone derivatives *via*l-proline/ Et_3_N-catalyzed aldol condensation and study of their antioxidant potential

**DOI:** 10.1016/j.mex.2023.102427

**Published:** 2023-10-12

**Authors:** Nur Rohman, Bayu Ardiansah, Antonius Herry Cahyana, Nurhayati

**Affiliations:** a Department of Chemistry, Faculty of Mathematics and Natural Sciences, Universitas Indonesia, Depok 16424, Indonesia; b Research Center for Marine and Land Bioindustry, National Research and Innovation Agency (BRIN), Pademangan, Jakarta 14430, Indonesia

**Keywords:** Azachalcone, Aldol condensation, l-proline/ Et_3_N, Antioxidant, Synthesis of azachalcones based 2-acetylpyridine

## Abstract

Chalcones, with two connected aromatic rings through an α,β-unsaturated carbonyl skeleton, display diverse biological roles like antimalarial, antibacterial, anticancer, and antioxidant activities. This research focuses on crafting azachalcone derivatives from 2-acetylpyridine and aromatic aldehydes using l-proline/Et_3_N as a catalyst. Refinements encompass catalyst dosage, solvents, temperature, and post-reaction treatments. The optimized approach employs l-proline (0.15 equiv.)/ Et_3_N (0.30 equiv.) at room temperature in methanol. Derivatives are successfully synthesized in moderate to favorable yields, akin to sodium hydroxide as the benchmark catalyst. Notably, antioxidant assessment via the DPPH method spotlights compound 2b and 2d (100 ppm concentration), showcasing significant antioxidant potency with inhibition percentages of 92.22 % and 74.41 %, respectively.

• l-proline/ Et_3_N is successful to use in aldol condensation reaction.
• Azachalcones based 2-acetylpyridine were successfully synthesized using the catalyst.
• Azachalcones showed antioxidant activity against DPPH radical.

## Graphical abstract

**Figure:**
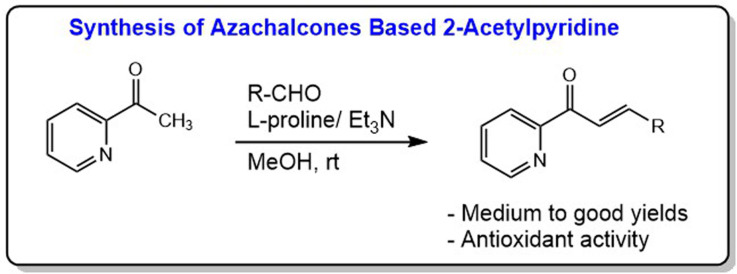

Specifications table

**Table:** 

Subject area:	Chemistry
More specific subject area:	Organic Chemistry
Name of your method:	Synthesis of azachalcones based 2-acetylpyridine
Name and reference of original method:	A novel and efficient direct aldol condensation from ketones and aromatic aldehydes catalyzed by proline–TEA through a new pathway. Tetrahedron 65 (2009) 4826–4833.
Resource availability:	The research was carried out at the Department of Chemistry, Faculty of Mathematics and Natural Sciences, Universitas Indonesia. All reagents were analytical grade and used without further purification. Suppliers are Merck, Sigma-Aldrich, and other local chemical companies based in Indonesia.

## Method details

Free radicals are molecules or molecular fragments that have one or more unpaired electrons in their outermost atomic or molecular orbitals. These unpaired electrons make them unstable, short-lived, and highly reactive. Due to their instability and high reactivity, free radicals attempt to reach a stable state by attracting electrons from other molecules or cells [1]. The ability of free radical molecules to attract electrons from other molecules could cause oxidative damage in the body. Oxidative stress occurred due to an imbalance between the production of free radicals and antioxidants in the body, triggered by an excess of free radicals and a deficiency of enzymatic and non-enzymatic antioxidants. Most importantly, an excess of free radicals could damage various biomolecules, including lipids, proteins, and DNA [2]. Antioxidants are a group of chemical compounds that could neutralize free radicals by donating electrons to the unpaired electrons of the free radicals, thereby reducing the oxidative effects of free radicals within cells [3]. Antioxidants play a crucial role in providing protection against damage caused by free radicals, as free radicals are implicated in various diseases such as aging, anemia, cancer, and cardiovascular diseases [4].

**Fig. 1:**
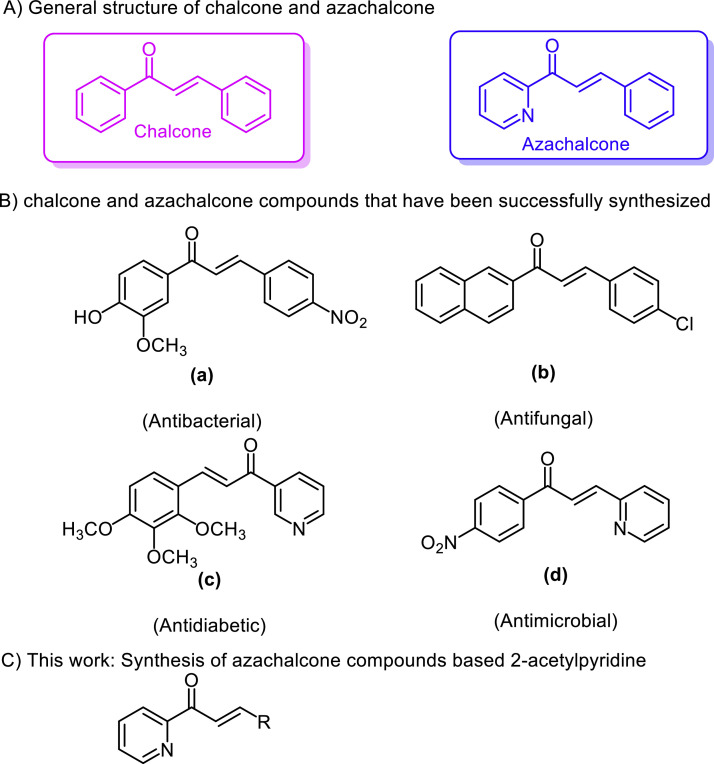
Design of the study.

**Scheme 1:**
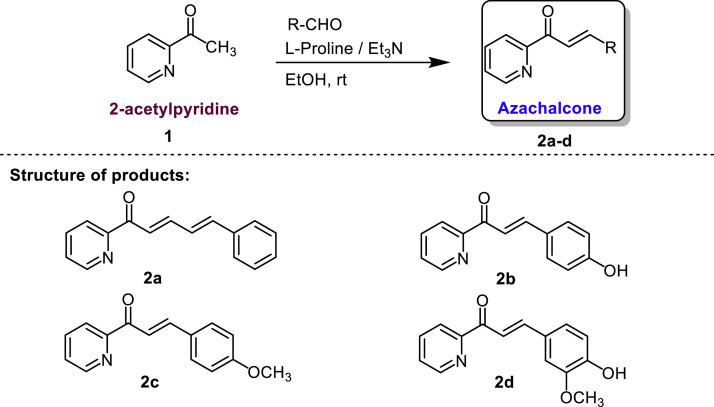
Synthesis of azachalcones from 2-acetylpyridine.

Chalcone, with the IUPAC name *(E)*−1,3-diphenylprop-2-en-1-one (Fig. 1A), is an important intermediate of flavonoid natural products [5]. It has a core structure consisting of two aromatic rings, connected with an α,β-unsaturated carbonyl group [6]. Various researches had been conducted to explore the bioactivities present in the chalcone derivatives, such as anti-inflammatory [7], anticancer [8], antibacterial [9], antioxidant [10], antiviral [11], antihypertensive, and antitumor activities [12,13]. One specific class of the chalcone is azachalcone. It is a chalcone compound with a nitrogen atom at the aromatic ring (Fig. 1A). This subclass has diverse biological activities, including antibacterial, antioxidant, and α-glucosidase enzyme inhibition activities [14,15]. Several chalcone compounds possessing bioactivities are depicted in Fig. 1B [15], [16], [17], [18]. With the advantages of chalcones, especially subclass azachalcone, we report here the synthesis of a series of azachalcone derivatives by reacting 2-acetylpyridine with various aromatic aldehydes, catalyzed by l*-*proline / triethylamine (Fig. 1C). The synthesized products (Scheme 1) were then evaluated as candidates of antioxidant.

The synthesis of azachalcone derivatives was carried out following the reported method with modification of the substrates [19]. Synthesis with l-proline/ Et_3_N catalyst: In a 10 mL round bottom flask, a mixture containing 2-acetylpyridine (1.0 mmol, 121 mg) and aromatic aldehyde (1.0 mmol) in methanol (2 mL) was added l-proline (0.15 mmol, 17 mg). Then, triethylamine (0.30 mmol, 31 mg) was added dropwise to the mixture. The reaction mixture was then stirred for 24 h at room temperature. Synthesis with NaOH catalyst: In a 10 mL round bottom flask, a mixture containing 2-acetylpyridine (1.0 mmol, 121 mg) and aromatic aldehyde (1.0 mmol) in methanol (2 mL) was prepared. A solution of sodium hydroxide (0.6 g in 3 mL ethanol) was added dropwise to the mixture with continuous stirring for 10 min. The reaction mixture was then stirred for 24 h at room temperature. The reaction was monitored using thin layer chromatography with *n*-hexane/ ethyl acetate as the mobile phase. After the reaction was over, the mixture was extracted with ethyl acetate to wash with water and brine. The combined organic phase was dried over anhydrous sodium sulfate, and then solvent was evaporated to obtain the crude product. Purification of the crude product was performed using column chromatography with a mixture of *n*-hexane/ ethyl acetate gave pure azachalcone **2a-d**.

**Scheme 2:**
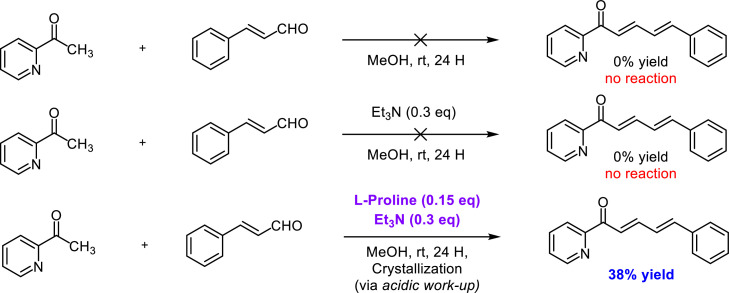
Initial trials in optimization process to produce compound 2a.

The optimization process aimed to discover the optimal conditions for synthesizing the target compounds (Scheme 2). 2-acetylpyridine and *trans*-cinnamaldehyde as model substrates were utilized in this endeavor. During the first optimization, it was acknowledged that the aldol condensation reaction in the synthesis of azachalcone is typically catalyzed by a base. Therefore, to exploit the basicity of 2-acetylpyridine (pK_b_ value = 11.32), we performed the reaction without any catalyst in methanol at room temperature for 24 h. Despite anticipating a successful reaction due to the substrate's autocatalysis process, the observed trial did not exhibit any reaction. This was evident from the lack of color change before and after the reaction. Subsequently, a second optimization process was conducted by enhancing the basicity of the reaction medium through the addition of triethylamine. However, this attempt also failed to yield any chemical reaction, as triethylamine was not potent enough to deprotonate the alpha hydrogen of 2-acetylpyridine. With no success in the first two trials, the third optimization process was initiated. This time, l-proline and triethylamine were combined as catalysts, followed by purification through an acidic work-up process and subsequent crystallization. The utilization of the l-proline/ Et_3_N combination resulted in a 38 % yield of the target product (**2a**). A clear visual indicator of the successful reaction was the significant color change from yellow to brown during the reaction. Building on the insights gained from the initial optimization, further refinement was pursued to identify the most favorable conditions for the aldol condensation reaction.

The subsequent optimization process was carried out by changing the treatment after the reaction (Table 1). The first extended optimization process was carried out without adding hydrochloric acid in the purification process after the reaction. Direct extraction of the reaction mixture with ethyl acetate and then purification by silica gel chromatography gave the product in 74 % yield. The second extended optimization process was conducted by changing the solvent from methanol to ethanol: water in a 1:1 ratio, followed by the same treatment as the first extended optimization, and a yield of 19 % was obtained. The third extended optimization process was performed by raising the temperature to 50 °C, followed by the same treatment as the first extended optimization, gave the product in 71 % yield. Based on the results of the extended optimization, the second entry obtained the highest yield of 74 %, indicating the best optimization with the treatment without adding acid (HCl), conducting extraction, using methanol as the solvent, and at room temperature. Addition of hydrochloric acid during the post-reaction treatment caused the protonation of pyridine moiety so that the product become water soluble (not extracted by ethyl acetate). Meanwhile, heating condition has a significant influence on the reaction by increasing kinetics energy of the reactants and more activating the catalyst [21]. However, in the heating condition, some unwanted spots were observed. Therefore, the best condition for the azachalcone synthesis is using l-proline (0.15 eq) and trimethylamine (0.3 eq) at room temperature for 24 h, without addition of hydrochloric acid in the post-reaction treatment.

**Table 1:** Results of the extended optimization process.


Entry	Temp. (℃)	Solvent	Post-reaction treatment	Yield (%)
1	rt	MeOH	Addition of HCl; crystallization	38
**2**	**rt**	**MeOH**	**Without addition of HCl; extraction**	**74**
3	rt	EtOH / H_2_O (1:1)	Same as entry 2	19
4	50	MeOH	Same as entry 2	71

The synthetic scope of this procedure was extended (Table 2). Other aldehydes such as 4-hydroxybenzaldehyde, 4-methoxybenzaldehyde, and vanillin, were employed in this reaction. We have also compared the catalytic activity of l-proline / Et_3_N with sodium hydroxide as the standard catalyst for aldol condensation. Under the optimized conditions, compound **2a** was obtained in 74 % yield using l-proline / Et_3_N as catalyst. Meanwhile, employing sodium hydroxide as catalyst for the same substrate, the product was afforded in slightly higher yield (80 %). Compounds **2b** and **2c** were obtained in low to medium yield using l-proline / Et_3_N. Unfortunately, product **2d** was obtained in trace under the optimized conditions. This is because triethylamine (Et_3_N) was consumed due to its reaction with the phenolic group, so that it cannot work synergistically with l-proline to catalyze the reaction. As expected, increasing the amount of triethylamine from 0.3 eq. to 1.3 eq. can improve the yield of **2d** In all cases, the azachalcone derivatives were obtained in lower yield using l-proline / Et_3_N catalyst than sodium hydroxide catalyst. However, this method highlighted the potency of l-proline / Et_3_N to be used as organocatalyst for a wide range of structural azachalcone derivatives. Table 2 summarizes the synthesis of azachalcone using various aromatic aldehydes in methanol solvent and stirring for 24 h at room temperature with variations of l*-*proline / Et_3_N and NaOH as catalysts.

**Table 2:** Summary of the synthesized products catalyzed by l-proline/Et_3_N and NaOH^a^.

Product	Structure	Yield (%)	
		L-Proline/Et_3_N	NaOH
2a		74	80
2b		30^b^	40
2c		16	49
2d		Trace; 24^b^	40

Note: a) Reaction scale of 1.0 mmol, using l*-*Proline (0.15 eq) / Et_3_N (0.30 eq) as well as NaOH b) l*-*Proline (0.15 eq) / Et_3_N (1.30 eq) yields 24 % for product **2d** and 30 % for product **2b**.

Here is the characterization data:

(2*E*,4*E*)−5-phenyl-1-(pyridin-2-yl)penta-2,4‑dien-1-one **(2a)**

The reaction of **1** (1.0 mmol, 121 mg) with *trans*-cinnamaldehyde (1.0 mmol, 132 mg), following the general procedure, gave the title compound **2a** in 74 % yield (174 mg) with l-proline / Et_3_N catalyst and 80 % yield (188 mg) with NaOH (0.6 g in 3 mL ethanol) catalyst. Yellow solid; Rf value 0.45 (hexane/ethyl acetate = 10/1); IR (KBr, disc) *ν_max_* 3060, 3027, 1661, 1596, 1577, 1354, 1029 cm^−1^; HR-MS (ESI-TOF) Calcd for C_16_H_14_NO [*M* + H*]*^+^ 236.1075, found 236.1068.

(*E*)−3-(4-hydroxyphenyl)−1-(pyridin-2-yl)prop‑2-en-1-one **(2b)**

The reaction of **1** (1.0 mmol, 121 mg) with 4-hydroxybenzaldehyde (1.0 mmol, 122 mg), following the general procedure, gave the title compound **2b** in 30 % yield (68 mg) with l-proline / Et_3_N or 40 % yield (90 mg) with NaOH (0.6 g in 3 mL ethanol) catalyst. Green solid; Rf value 0.25 (hexane/ethyl acetate = 3/1); IR (KBr, disc) *ν_max_* 3320, 3082, 3064, 1667, 1593, 1586, 1347 cm^−1^; HR-MS (ESI-TOF) Calcd for C_14_H_12_NO_2_ [*M* + H*]*^+^ 226.0868, found 226.0884.

(*E*)−3-(4-methoxyphenyl)−1-(pyridin-2-yl)prop‑2-en-1-one **(2c)**

The reaction of **1** (1.0 mmol, 121 mg) with 4-methoxybenzaldehyde (1.0 mmol, 136 mg), following the general procedure, gave the title compound **2c** in 16 % yield (38.2 mg) with l*-*proline / Et_3_N (0.3 mmol) catalyst and 49 % yield (117 mg) with NaOH (0.6 g in 3 mL ethanol) catalyst. Yellow crystal; Rf value 0.40 (hexane/ethyl acetate = 5/1); IR (KBr, disc) *ν_max_* 3075, 3057, 2845, 1668, 1594, 1580, 1350, 1270 cm^−1^; HR-MS (ESI-TOF) Calcd for C_15_H_14_NO_2_ [*M* + H*]*^+^ 240.1024, found 240.1036.

(*E*)−3-(4‑hydroxy-3-methoxyphenyl)−1-(pyridin-2-yl)prop‑2-en-1-one **(2d)**

The reaction of **1** (1.0 mmol, 121 mg) with vanillin (1.0 mmol, 152 mg), following the general procedure, gave the title compound **2d** in trace with l-proline / Et_3_N (0.3 mmol) catalyst, or 24 % yield (61 mg) with l*-*proline / Et_3_N (1.3 mmol) catalyst, or 40 % yield (102 mg) with NaOH (0.6 g in 3 mL ethanol) catalyst. Yellow solid; Rf value 0.29 (hexane/ethyl acetate = 3/1); IR (KBr, disc) *ν_max_* 3290, 3062, 3047, 2830, 1666, 1592, 1581, 1265, 1020 cm^−1^; HR-MS (ESI-TOF) Calcd for C_15_H_14_NO_3_ [*M* + H*]*^+^ 256.0973, found 256.0980.

**Fig. 2:**
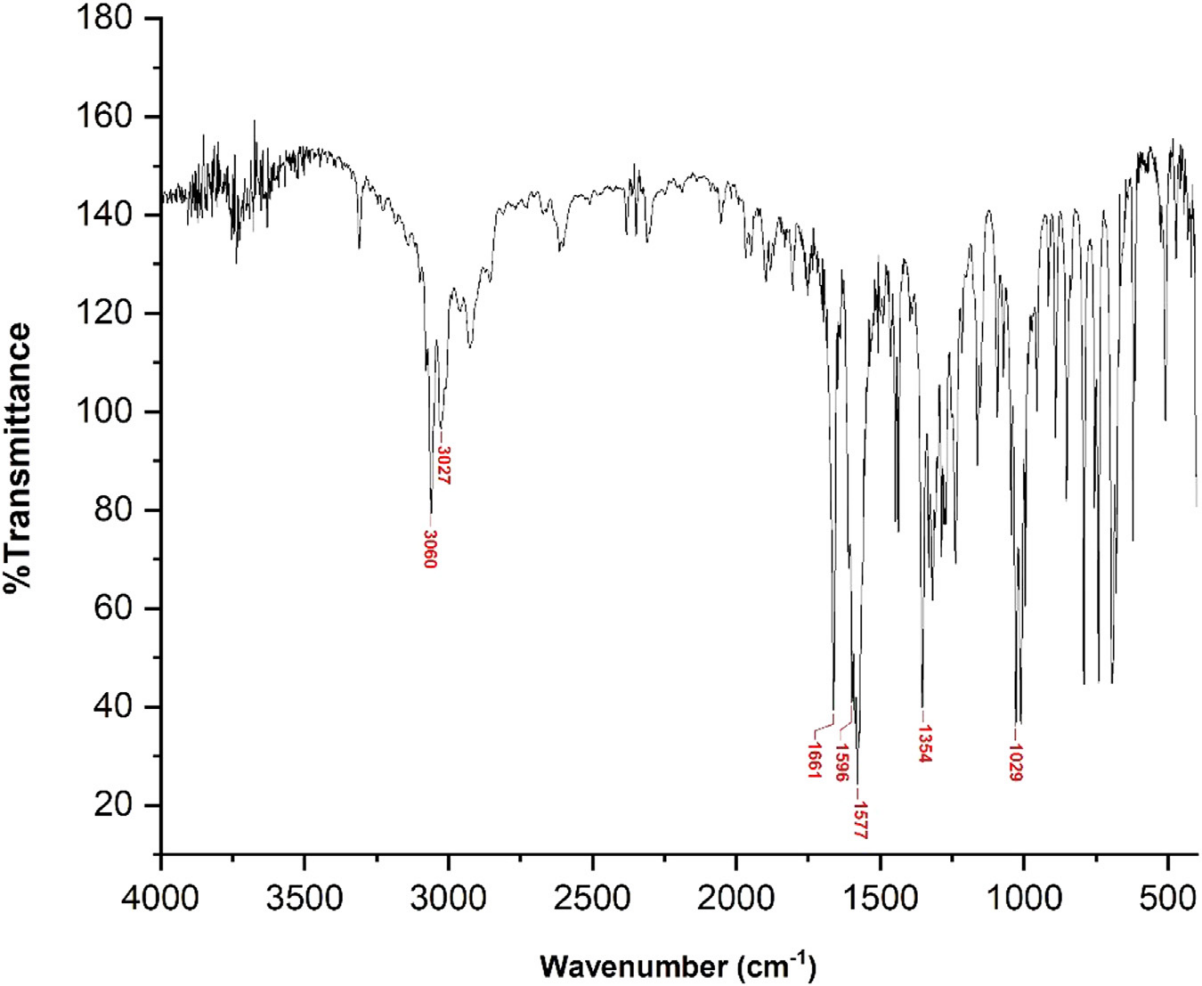
FTIR spectrum of compound 2a.

**Fig. 3:**
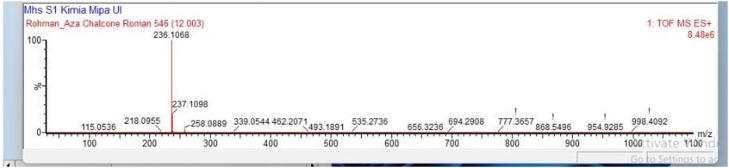
HRMS spectrum of compound 2a.

The structures of the synthesized azachalcones **2a-d** were confirmed through FTIR and HRMS analysis. Based on the FTIR analysis, the formation of azachalcones **2a-d** was confirmed through the absorption peaks, consistently showing vibrations in the C—H *Sp*^2^ aromatic stretching at 3027 – 3082 cm^−1^, C=O stretching at 1661 – 1668 cm^−1^, C=N stretching at 1592 – 1596 cm^−1^, and C-H *Sp*^2^ stretching at 1577 – 1586 cm^−1^, for each compound. In addition to FTIR, HR-MS analysis was conducted using TOF/MS equipped with ESI as the ionization source. Each sample was analyzed in positive mode based exclusively on LC retention time and high-resolution mass spectra. The HR-MS mode was performed in a hybrid quadrupole and time-of-flight analysis in positive mode. From experimental results, the [*M* + *H*] values of azachalcones **2a-d** are very close compared to the theoretical values showing consistent results. For instance of product characterization, compound **2a** exhibited IR absorption peaks at 3060 and 3027 (C—H *Sp*^2^ aromatic stretching), 1661 (C = O stretching), 1596 (C=N stretching), 1577, 1354, and 1029 cm^−1^ (Fig. 2). Meanwhile, from HR-MS, the theoretical value for compound **2a** [*M* + *H*]^+^ was 236.1075, while the experimental result was found to be 236.1068 (Fig. 3).

**Scheme 3:**
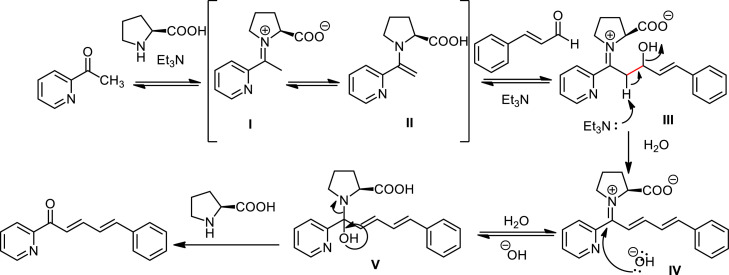
Mechanism of the l-proline/Et_3_N catalysis in aldol condensation reaction.

Despite the choice of catalyst depends on the specific substrates, the utilization of organocatalyst such as l-proline provide some advantages: (1) Organocatalysts typically operate under milder reaction conditions, including lower temperatures and neutral pH, which can be beneficial for substrates that are sensitive to harsher conditions, (2) Organocatalysts are compatible with a wider range of functional groups, which can be advantageous when dealing with complex molecules, (3) Organocatalysts can be effective at lower catalyst loadings, which can reduce the overall cost of the reaction. In general, the formation of *(E)-*α,β-unsaturated carbonyl compounds catalyzed by l-proline / Et_3_N proceeds through the enamine mechanism (Scheme 3). l-proline forms an enamine compound at the carbonyl group of the 2-acetylpyridine compound, resulting in intermediates **I** and **II**. Once intermediate **II** is formed, aldol reaction takes place via the enamine, leading to the formation of β‑hydroxy enamine **III**, which then undergoes a dehydration process to produce intermediate **IV**. The function of Et_3_N in this reaction mechanism is to abstract the alpha hydrogen from intermediate **III,** facilitating the elimination of the hydroxyl group and forming a new double bond. After l-proline leaves the intermediate **V** compound, the α,β-unsaturated carbonyl is formed.

The synthesized compounds of azachalcone derivatives were subjected to antioxidant testing using the 1,1-diphenyl-2-picrylhydrazyl (DPPH) technique, following the previous research with some modifications [20]. 10 mg of DPPH was dissolved in 100 mL of ethanol to prepare the stock solution. The absorbance of the DPPH stock solution was measured at a wavelength of 517 nm. In a test tube, 2 mL of DPPH solution was mixed with 2 mL of the synthesized compound sample. After that, the test tubes were kept in the dark for 30 min. The absorbance was then determined at 517 nm. The testing was performed at a single concentration point, which was 100 ppm, and repeated 3 times. The percentage of inhibition was measured using the equation: Inhibition (%) = [(C-S)/C] × 100 %. Where: C = Absorbance of control; *S* = absorbance of sample.

The antioxidant evaluation was conducted using the DPPH Radical Scavenging method, where the DPPH compound was used as a stable free radical that captures a proton from the antioxidant compounds being tested for bioactivity [22]. Compounds with high antioxidant properties will rapidly change the color of the solution from purple to yellow. From the bioactivity testing results as antioxidants for the four Aza-chalcone compounds in Table 3, it was found that the presence of a hydroxyl group (-OH) on the benzene ring increases the inhibition value due to the ease of proton donation from the hydroxyl group (-OH) to the free radical compound. On the other hand, the presence of a methoxy group (–OCH_3_) reduces the inhibition value. This is evidenced by the decrease in the% inhibition value from compound **2b**, which has only a hydroxyl group (-OH), to compound **2d**, which has both a hydroxyl group (-OH) and a methoxy group (–OCH_3_). In the antioxidant evaluation results, it was found that three compounds had inhibitory values at a concentration of 100 ppm greater than 50 % (compounds **2b, 2d, 2a**), while one compound had an inhibitory value less than 50 % (compound **2c**). As a newly synthesized compound, the α,β,γ,δ-unsaturated azachalcone (**2a**) exhibited moderate inhibitory activity against DPPH.

**Table 3:** Antioxidant activity of the tested solution at 100 ppm.

Product	Structure	Inhibition of DPPH (%)
2a		60.74 ± 0.22
2b		92.22 ± 0.19
2c		42.51 ± 0.14
2d		74.41 ± 0.20

In conclusion, the synthesis process of azachalcone compounds, starting from optimization, azachalcone compound synthesis, to antioxidant potential testing using the DPPH method, has been carried out. In the optimization process, the best synthesis result was obtained by adding l-proline (0.15 eq) / Et_3_N (0.3 eq) catalyst in methanol solvent, stirred for 24 h at room temperature, and followed by post-reaction treatment without the addition of acidic compounds and extraction process. In the synthesis process of azachalcone compounds, 2-acetylpyridine compound was reacted with four variations of aromatic aldehydes, namely trans-cinnamaldehyde, 4-hydroxybenzaldehyde, vanillin, and 4-methoxybenzaldehyde, using l-proline / Et_3_N and NaOH catalysts. The best synthesis result was obtained using trans-cinnamaldehyde as the aromatic aldehyde variation, with a yield of 74 % using l*-*proline / Et_3_N catalyst and 80 % using NaOH catalyst. In the antioxidant potential testing, compounds with hydroxyl (-OH) groups showed higher activity compared to compounds without hydroxyl (-OH) groups. Compounds **2b** and **2d** were the most active antioxidants in this series, exhibiting antioxidant activities with inhibition percentages of 92.22 % and 74.41 %, respectively. Additionally, compounds with methoxy (–OCH_3_) groups showed a decrease in antioxidant activity compared to compounds without methoxy (–OCH_3_) groups. This research clearly identifies compounds **2b** and **2d** as interesting candidates for further development as antioxidants.

## CRediT authorship contribution statement

**Nur Rohman:** Investigation, Data curation, Writing – original draft. **Bayu Ardiansah:** Conceptualization, Methodology, Validation, Supervision, Writing – review & editing. **Antonius Herry Cahyana:** Supervision, Writing – review & editing. **:** Data curation, Investigation.

## Data Availability

No data was used for the research described in the article.
